# Nonverbal synchrony of head- and body-movement in psychotherapy: different signals have different associations with outcome

**DOI:** 10.3389/fpsyg.2014.00979

**Published:** 2014-09-05

**Authors:** Fabian Ramseyer, Wolfgang Tschacher

**Affiliations:** Department of Psychotherapy, University Hospital of PsychiatryBern, Switzerland

**Keywords:** nonverbal synchrony, embodiment, psychotherapy, motion energy analysis, head movement, body-movement, process-outcome research

## Abstract

**Objective:** The coordination of patient’s and therapist’s bodily movement – nonverbal synchrony – has been empirically shown to be associated with psychotherapy outcome. This finding was based on dynamic movement patterns of the whole body. The present paper is a new analysis of an existing dataset (Ramseyer and Tschacher, 2011), which extends previous findings by differentiating movements pertaining to head and upper-body regions.

**Method:** In a sample of 70 patients (37 female, 33 male) treated at an outpatient psychotherapy clinic, we quantified nonverbal synchrony with an automated objective video-analysis algorithm (motion energy analysis). Head- and body-synchrony was quantified during the initial 15 min of video-recorded therapy sessions. Micro-outcome was assessed with self-report post-session questionnaires provided by patients and their therapists. Macro-outcome was measured with questionnaires that quantified attainment of treatment goals and changes in experiencing and behavior at the end of therapy.

**Results:** The differentiation of head- and body-synchrony showed that these two facets of motor coordination were differentially associated with outcome. Head-synchrony predicted global outcome of therapy, while body-synchrony did not, and body-synchrony predicted session outcome, while head-synchrony did not.

**Conclusion:** The results pose an important amendment to previous findings, which showed that nonverbal synchrony embodied both outcome and interpersonal variables of psychotherapy dyads. The separation of head- and body-synchrony suggested that distinct mechanisms may operate in these two regions: Head-synchrony embodied phenomena with a long temporal extension (overall therapy success), while body-synchrony embodied phenomena of a more immediate nature (session-level success). More explorations with fine-grained analyses of synchronized phenomena in nonverbal behavior may shed additional light on the embodiment of psychotherapy process.

“Relationships are not created by the brain; rather, the brain was created to serve relationships.”

(Baumeister, 2012, p. 136)

## INTRODUCTION

Social interaction is a core ingredient of human existence and people have a basic need to belong to other people (Baumeister and Leary, 1995; Baumeister, 2012). The motive for connection – called communion in interpersonal theory (Horowitz et al., 2006) – is observable in most forms of social exchange and interpersonal behavior. The mechanisms involved in this complex and dynamic interplay are manifold, and traditionally, a basic distinction between verbal and nonverbal communication channels has been made. For a long time, nonverbal behavior has been recognized as an important facet of social interaction (Knapp et al., 2013), and various efforts have been made to use this often overlooked source of information. These attempts have been most evident in truth verification (e.g., Ekman and Friesen, 1974; DePaulo and Rosenthal, 1979; Vrij and Semin, 1996; DePaulo et al., 2003; Duran et al., 2013). For instance, the Supreme Court of Canada has recently ruled that judges and jurors must view a witness to “adequately evaluate body language, facial expressions, and other indicators of credibility” (Porter et al., 2012).

Observable manifestations of nonverbal behavior are best described within the framework of embodiment (Oberzaucher and Grammer, 2008; Storch et al., 2010; Tschacher and Bergomi, 2011), and the association between emotion and motion is also well captured from the stance of embodied cognition (Niedenthal, 2007). In this paper, we will focus on phenomena of embodiment in psychotherapy dyads: a previous study on nonverbal behavior in psychotherapy (Ramseyer and Tschacher, 2011) has identified nonverbal synchrony – the coordination of patient’s and therapist’s body-movement – as an indicator of embodied processes in the therapy dyad. Here, we aim to extend this finding by looking more closely at different regions of the body and how their coordination may relate to indices of success in therapy. We assume that the differentiation between body regions will provide additional insight into the dynamics of nonverbal exchange.

Traditionally, research in the domain of nonverbal communication has strongly focused on signals transmitted by the face (de Gelder, 2009). The human nervous system has specialized subsystems that are fine-tuned for such signals: certain regions of the brain are involved when analyzing facial features for the purpose of, e.g., face recognition (fusiform gyrus: Kanwisher et al., 1997), or decoding of emotional signals in facial displays (amygdala: Morris et al., 1998). Similarly, however, selective cortical areas for visual processing of the human body have been identified (extrastriate body area: Downing et al., 2001; Koningsbruggen et al., 2013). Recent neurophysiological evidence implies that face and body perception may rely on different neurocortical systems (Meeren et al., 2013; Van den Stock et al., 2014). The larger part of these processes occur outside conscious awareness (Whalen et al., 1998) both for the encoding as well as the decoding of actions (e.g., micro-expressions: Matsumoto and Willingham, 2006). There is a consensus in the popular literature on “body language” that body parts farther away from the head (e.g., a person’s legs and feet) are progressively less under conscious control (e.g., Pease and Pease, 2006; Reiman, 2007; Goman, 2008), and would therefore betray a person’s “hidden intentions.” Yet such assertions – to our knowledge – have never been tested empirically.

Generally, nonverbal communication uses dynamical information, not only static features. Therefore, movement dynamics is a core facet in the nonverbal domain. For example, the accuracy of detecting facial emotion in movies (i.e., with visible movements of the face) is significantly higher than in still photographs (Brick et al., 2009). Accordingly, body motion detection and interpretation are crucial for social perception (Grèzes and de Gelder, 2009). Thus, the whole body (not just the face) may be viewed as the essential “signaling device” in emotional processing (de Gelder, 2006), and such signals are prime sources of social information.

From an evolutionary point of view (Boone and Buck, 2003), it is vitally important to accurately navigate in social surroundings, because “the basic discrimination of friend and foe likely was one of the earliest interpersonal judgments to evolve” (Williams and Mattingley, 2006). This implies that apart from the accurate detection and decoding of nonverbal information, relevant implications for the interpersonal consequences of an encounter should also be registered and incorporated into the behavioral and emotional responses of an individual. The association between emotional experience and nonverbal behavior is tightly linked (e.g., Niedenthal et al., 2010; Lausberg and Kryger, 2011; Dael et al., 2012). Grahe and Bernieri (1999, p. 265) stated that “rapport is primarily a physically manifested construct; it is a construct that is visible at the surface and readily apparent. (...) In other words, rapport simply may be visible.” In neurobiological terms, sensorimotor loops and the mirror-neuron system are able to transform the primary perception of a partner’s acts into an interpretation of the partner’s emotions and intentions. Most of these processes occur very fast and outside of conscious awareness (Tamietto and de Gelder, 2010). This is also true for the domain of gross body-movement and locomotion detection (Blake and Shiffrar, 2007). The specificity of movement detection is evident early in life (Baldwin et al., 2001) and it is highly relevant for any kind of human social interaction (Burgoon, 1994). Findings from patients suffering from autism spectrum disorders highlight the social consequences of inaccurate, delayed, or missing nonverbal processing (Klin et al., 2009).

Research on the core ingredients of psychotherapy has pointed to a significant role of the therapeutic alliance: the relationship quality between therapist and patient is one of the best empirically supported predictors of therapy outcome (Horvath et al., 2011; Flückiger et al., 2012). The alliance is considered to have several components such as mutual sympathy, pursuing shared goals, and the overcoming of resistance to change. Psychotherapists in practice always regard their own and their patients’ nonverbal behavior (Hall et al., 1995). Recently, however, this topic has almost disappeared from view in psychotherapy research, as evidenced by the lack of references to nonverbal behavior in the latest edition of the Handbook of Psychotherapy and Behavior Change (Lambert, 2013). At the same time, various new approaches for the analysis of nonverbal behavior have appeared in social and clinical psychology (Frey and von Cranach, 1973; Bänninger-Huber, 1992; Altorfer et al., 2000; Boker and Rotondo, 2002; Grammer et al., 2003; Brick and Boker, 2011; Lavelle et al., 2012). Work on mimicry/imitation focused mainly on directly observable and quantifiable (body) movement behaviors (e.g., foot shaking, face rubbing). Recent, highly sophisticated research has addressed head movement dynamics (Boker et al., 2009), showing that the dynamics was the relevant factor that influenced behavior in participants (Boker et al., 2011). This is also found in research on man-machine interfaces: avatars that mimic the head movements of an interaction partner are evaluated more favorably than avatars that do not display such imitative head movements (Bailenson et al., 2004, 2008; Reidsma et al., 2010). Along a similar line, body sway has been shown to become entrained in everyday face-to-face communication (Higo et al., 2012).

Thus, such advances in different fields suggest that disentangling of different body-movement regions may be a next step for research on nonverbal communication in the context of psychotherapy (Henry et al., 2012). We will base these new analyses on a database that was established by a previous study of psychotherapy dyads (Ramseyer and Tschacher, 2011). In the present article we will be focusing on nonverbal signals transmitted by the face (head movement) in contrast to nonverbal signals transmitted by the body (movement of the upper torso and hands) and how these signals relate to measures of success in psychotherapy. Our approach is mainly descriptive and exploratory – we report the extent of movement in the different body regions and the coordination (i.e., the nonverbal synchrony) of patients and therapists based on movement in these regions. Our expectation was that the nonverbal variables would differ in their associations with therapy outcome measures.

## MATERIALS AND METHODS

### SAMPLE

The present dataset is a subsample of previously published data (Ramseyer and Tschacher, 2011), which consisted of psychotherapy sessions that were randomly drawn from the entire video-recorded data (*N* > 5000 recordings) of the outpatient center of the University of Bern, Switzerland. We randomly selected one single session of each dyad of the previous sample. This resulted in a total of *N* = 70 sessions of psychotherapy from 37 female and 33 male same-sex dyads (mean age 36.5 years, SD = 10.2, all white Caucasian European ethnicity). The sample contained 33 sessions from the initial phase and 37 sessions from the final phase of the respective patient’s therapy. Patients belonged to the following main diagnostic groups: 34% anxiety disorders, 29% affective disorders, 37% other diagnoses (11.4% adjustment disorders, 8.6% personality disorders, 17% other disorders). Comorbidity was predominantly found in anxiety disorders (58% comorbid patients) and affective disorders (24%). These percentages are closely representative of the complete database of the outpatient center of *N* = 838 cases, where 35.1% of patients were diagnosed with anxiety disorders, 24.8% affective disorders, 10.5% adjustment disorder, 4.3% eating disorders, and 15% with no axis-1 disorder. All clinical diagnoses were assessed before initiation of therapy using the Structured Clinical Interview (SCID; Wittchen et al., 1997) for the *Diagnostic and Statistical Manual of Mental Disorders* [DSM-IV; American Psychiatric Association (APA), 1994].

Mean psychotherapy duration per patient of the present sample was 38.1 sessions (SD = 22.1, range 8–126). Recording of therapy sessions was part of routinely ongoing research activity and quality assurance. Sessions were generally conducted once a week, each lasting 50 min on average. Patients and therapists sat in comfortable chairs facing each other with an angle of ∼110° at a distance of 1.5–2.5 m. Administration of psychotherapy and recording of sessions was independent of the research reported here, and took place before the formulation of research hypotheses, from 1998 to 2004. At the time of recording, patients and therapists were informed about further scientific use of their data and gave informed consent according to Swiss ethical regulation policies. For reasons of comparability and standardization, we analyzed only sessions from same-sex dyads, as was done in the previous analysis (Ramseyer and Tschacher, 2011). The limitation to same-sex dyads was based on research showing that mixed-gender dyads displayed lower nonverbal synchrony (Grammer et al., 1998). Only the first 15 min of any therapy session were chosen for our study. This limitation was put in place because we regarded only interaction sequences where dyads remained seated throughout, i.e., 15 min segments of psychotherapy where patients and therapists exclusively engaged in speaking/listening activity. Instances of, e.g., use of a flip chart or similar device, which implied leaving one’s chair, were excluded from analyses.

### MOTION ENERGY ANALYSIS

**FIGURE 1:**
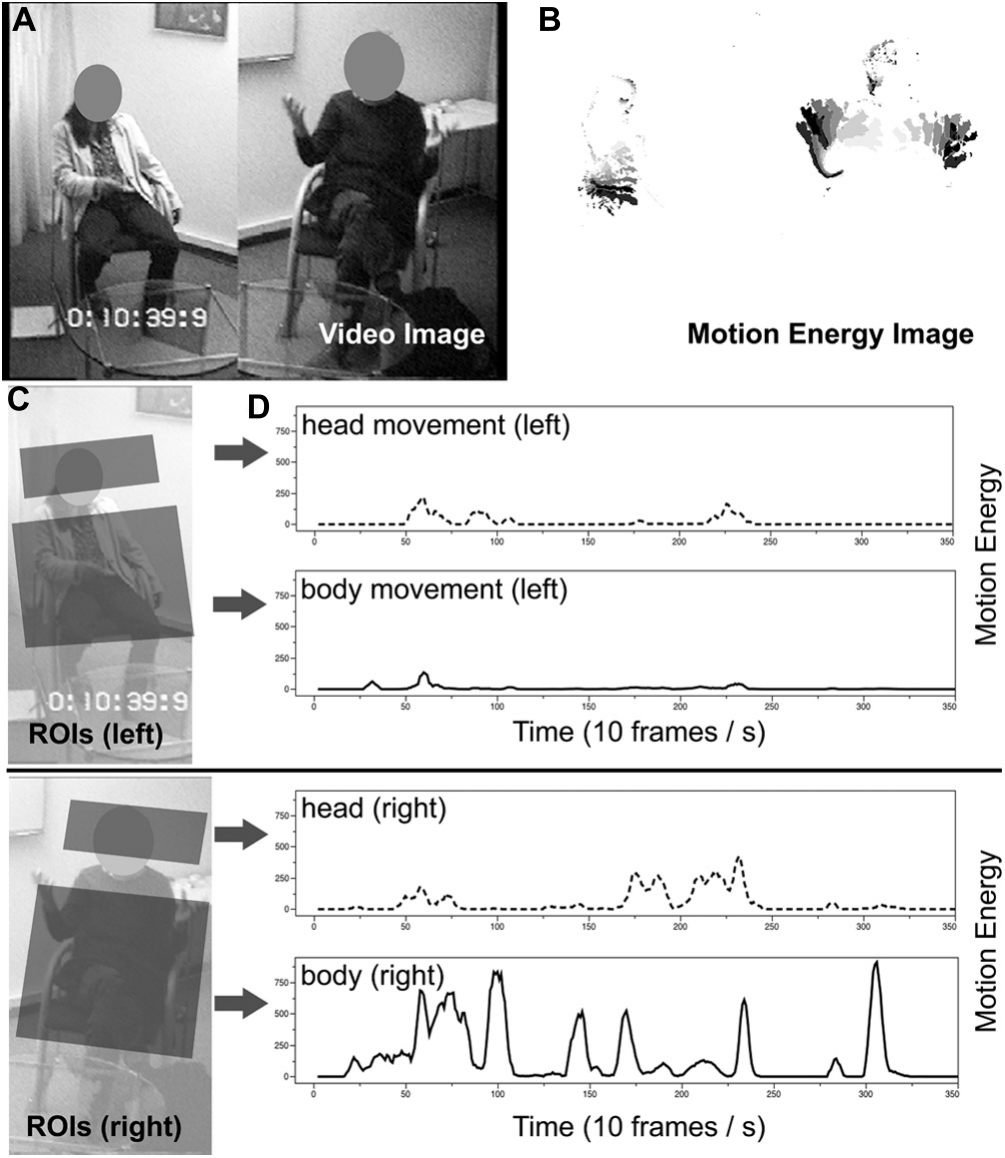
**(A)** Original video recording; **(B)** Difference-image derived by motion energy analysis; **(C)** Definition of regions of interest; **(D)** Time-series (35 s shown) of raw movement for each defined region of interest.

Motion energy analysis (MEA; Ramseyer, 2014) is a theory-free, objective, and fully automated computer program designed to quantify movement behavior in digital video recordings. Motion energy is defined as differences in gray-scale pixels between consecutive video-frames (frame-differencing: Grammer et al., 1997, 1999; Ramseyer and Tschacher, 2006; Nagaoka and Komori, 2008; Altmann, 2011; Paxton and Dale, 2013). Detection of frame-by-frame change allows an objective quantification of movement occurring in spatially pre-defined regions of interest (ROI’s; see **Figure 1C**). MEA thus generates time-series of raw pixel-change within a ROI that were filtered and corrected prior to further analyses (see **Figure 1D**). Details of the processing of raw signals are described in Grammer et al. (1999), further information on MEA is provided in Ramseyer and Tschacher (2011) and may be accessed online (www.psync.ch).

### NONVERBAL SYNCHRONY

Nonverbal synchrony was conceived as a dynamic quality capturing movement characteristics irrespective of the type of posture displayed in a ROI. Nonverbal synchrony thereby constitutes an objective quantification of the *dynamic* movement characteristics displayed by patient and therapist.

To compute synchrony, the time-series of motion energy (**Figure 1D**) were cross-correlated (Boker et al., 2002; Derrick and Thomas, 2004) in window segments of 1 min duration, thus taking into consideration the non-stationarity of movement behaviors. Movements were cross-correlated with time-lags up to ±5 s, in order to allow for exactly simultaneous synchronization (lag of 0 s) and delayed synchronization (lags up to ±5 s). Absolute values of cross-correlation were aggregated over the entire interval of 15 min in each session.

### DIFFERENTIATION OF REGIONS OF INTEREST

Separate regions for head movement and upper-body movement were chosen (see **Figure 1**). Previous work in the psychotherapy setting indicated differences between head movement and body-movement (Fretz, 1966), which was confirmed in a more recent study with schizophrenia patients (Kupper et al., 2010). Two ROIs were defined per participant: the head region covered the head including the neck and thus contains all head and neck movements; the body region covered the upper-body from the chair’s seating-base upward and the arms. Both ROIs are shown in **Figure 1C**. Boundaries of ROIs were defined such that a zone of non-contact resulted between head and body. This was done in order to minimize possible region-crossings, i.e., movement from one region being erroneously registered in the other region. The most frequent example of region-crossing is self-touch of the facial region by a hand. Spontaneous facial self-touch occurs frequently (Nicas and Best, 2008), and was found to serve emotion-regulative purposes (Grunwald et al., 2014), and to entail notable effects on social impression formation (Harrigan et al., 1987). In the present analysis, we were not specifically interested in self-touch to the facial region, but in overall movement of head- and body-regions. We addressed this possible confounding aspect by simply regarding facial-touch as an instance of head-movement. This simplification/generalization may be considered conservative, because it attenuates the differentiation between head- and body-movement. At the level of statistical testing, we evaluated all synchrony-outcome associations either with or without partialling out the effect of the other region.

### SYNCHRONY VERSUS PSEUDOSYNCHRONY

A final step in quantifying nonverbal synchrony is to rule out that the detected movement synchrony may be spurious. We therefore corrected for random contingencies between the two movement streams of patient and therapist. In early research on interactional synchrony, a debate addressed the genuineness of Condon and Ogston’s (1966) findings (McDowall, 1978; Gatewood and Rosenwein, 1981). We acknowledge this critical consideration of synchrony findings by implementing a statistical mechanism that prevents false–positive detection of synchrony in psychotherapy sessions. To accomplish this, for each therapy session, we generated *N* = 100 surrogate datasets by shuﬄing the genuine data. In order to not destroy the microstructure of movement bursts, we shuﬄed each time-series windows-wise: the original structure inside one window remains intact, but due to shuﬄing of a window’s position, it is paired with another window from a different time in the therapy. For example, the motion energy values of the therapist’s behavior from the first minute may be aligned with the patient’s movements from the ninth minute of the same session. The significance of observed movement synchrony in comparison with chance levels of synchrony is then determined by how much the genuine cross-correlation coefficients departed from the mean shuﬄed coefficients (Ramseyer and Tschacher, 2010).

### MEASURES OF PSYCHOTHERAPY OUTCOME

Two types of outcome measures were used in this study. They captured change from different time-perspectives, which allowed both the quantification of session-level change – called micro-outcome – as well as overall therapy outcome – called macro-outcome (Ramseyer et al., 2014). The differentiation into micro- and macro-outcomes is not to be confounded with the level of evaluation used in, e.g., psychiatric assessments (Tomba and Bech, 2012), where the initial clinical judgment is called “macro-analysis” and a detailed analysis of symptoms is labeled “micro-analysis.” The distinguishing feature of the outcome measures employed in the present study thus lies in their temporal extension: some events in the therapy process may extend to outcome at the session level, whereas other events may have an impact on the outcome of the whole treatment. Therefore, different temporal dynamics are captured by micro- and macro-outcome.

### MICRO-OUTCOME

Post-session questionnaires were administered after the termination of each single therapy session as part of routine assessments. Patient (BPSR-P) and therapist (BPSR-T) versions of the *Bern Post-Session Report* (Flückiger et al., 2010) are self-report measures comprised of 22 (BPSR-P) and 27 (BPSR-T) items loading on five factors that were determined in previous factor analyses (Tschacher et al., 2007). Two factors captured the patient’s view of core properties of therapy process: patient’s alliance (exemplary item, “My therapist and I get along well”) and patient’s self-efficacy (“I feel more capable of solving my problems”). Other factors reflected the therapist’s perspective on alliance (therapist’s alliance: “Today, I felt comfortable with the patient”) and on the interventions implemented by the therapist; the interventions factors were not considered in the present analysis. Internal consistency of BPSR scales ranged from 0.74 to 0.88 as reported by Flückiger et al. (2010). As an extension for the present analyses in this sample, we constructed an additional factor based on three BPSR-T items that captured the therapist’s assessment of a patient’s resistance (“I find this to be an interactionally difficult patient”; “Did the patient show signs of being observant and reactive?”; “Did you notice patient’s resistance during conversation?”; Cronbach alpha = 0.82). These three items are part of the nine-item therapist alliance rating. We decided to also focus on this facet of problematic/oppositional behavior in the therapeutic relationship because we were interested in its association to head- and body-synchrony.

### MACRO-OUTCOME

The overall success of therapies was estimated with direct measures of success: patient self-report questionnaires assessing the amount of change caused by psychotherapy were applied once, at termination of a therapy course. In addition to these direct (retrospective) measures of success, further self-report questionnaires administered before and after therapy had been used. Here we report only direct measures of success as indicators of the macro-outcome of treatment (Michalak et al., 2003; Flückiger et al., 2007). Indirect pre-to-post outcome measures yielded lower associations with both head- and body-synchrony.

#### Goal attainment scaling

Goal attainment scaling (Cardillo and Smith, 1994) assesses to what extent the individual treatment goals explicitly defined at the beginning of therapies were reached. Assessments were performed by patients at the end of therapies. 7-point Likert scales are used, on which higher scores indicate greater goal attainment. The scores used here range from deterioration (-2: most unfavorable outcome thought likely) to no change (0: less than expected success with treatment) to various levels of improvement (4: best anticipated success with treatment). Cardillo and Smith (1994) reported inter-rater reliabilities of 0.87 and 0.71 for independent judges of GAS.

#### Changes in experiencing and behavior

The VEV [questionnaire to assess changes in experiencing and behavior (Veränderungsfragebogen des Erlebens und Verhaltens)] is a self-report measure used to assess the experienced changes and behavioral changes that are attributed to therapy. In Willutzki’s (1999) version, patients indicate in 27 items using a 7-point Likert scale to what extent their life has changed compared to a time-point directly before therapy (e.g., “Compared with the time prior to initiation of therapy, I feel more relaxed/more tense”). The measure provides a global index of overall improvement. Zielke and Kopf-Mehnert (2001) reported an internal consistency of 0.98 and test–retest reliability of 0.61 over a 8 week period.

## RESULTS

### INDIVIDUAL-LEVEL CHARACTERISTICS: BASIC MOVEMENT PARAMETERS

We begin with findings pertaining to individual movement parameters. A consistent pattern of movement activity was found: the relative amount of movement was expressed as percentage of time with above-threshold movement. Head-movement (PAT = 28.84%; TH = 33.92%) was higher than body-movement (PAT = 15.60%; TH = 21.34%) both in patients [*t*(69) = 15.79; *p* < 0.0001; *d* = 2.09] and in therapists [*t*(69) = 14.63; *p* < 0.0001; *d* = 1.60]. Female and male patients showed significant differences in their basic movement characteristics: female patients moved their heads more than male patients [*F* = 30.85%; *M* = 26.56%; *t*(69) = 2.89; *p* = 0.005; *d* = 0.70], while body-movement was similar for patients of both sexes [*F* = 16.09%; *M* = 15.07%; *t*(69) = 0.69; *p* = 0.508; *d* = 0.17]. Therapists showed a similar pattern, however, differences between male and female therapists were lower (and insignificant) in comparison to patient differences, both in head regions [*F* = 34.97%; *M* = 32.84%; *t*(69) = 1.24; *p* = 0.221; *d* = 0.30] and in body regions [*F* = 21.36%; *M* = 21.32%; *t*(69) = 0.02; *p* < 0.982; *d* = 0.01]. The three diagnostic groups were not significantly different in their basic movement parameters (see **Table 1**).

**Table 1:** Global movement parameters (mean percentage of movement) for head and body regions.

Diagnostic group	Female		Male		Both sexes	
	Head	Body	Head	Body	Head	Body
Affective disorders	31.65	17.40	25.42	14.50	28.68	16.02
Anxiety disorders	30.39	16.99	26.82	16.67	28.53	16.82
Other diagnoses	30.61	14.29	27.35	13.55	29.25	13.98

### DYAD-LEVEL CHARACTERISTICS: NONVERBAL SYNCHRONY

Significance of synchrony over pseudosynchrony was found for both ROIs and across all diagnostic groups. The amounts of nonverbal synchrony differed along the following lines: head-synchrony was higher than body-synchrony [0.089 versus 0.084; *t*(69) = 2.51; *p* = 0.014; *d* = 0.33]; no difference in terms of sex or diagnosis was found (all *p*s > 0.35). The comparison with pseudosynchrony indicated that the magnitude of the synchony-versus-pseudosynchrony difference was much higher in head-synchrony [*t*(69) = 6.03; *p* < 0.0001; *d* = 0.74; medium to high effect-size] than in body-synchrony [*t*(69) = 2.17; *p* < 0.05; *d* = 0.20; low effect-size].

### ASSOCIATIONS BETWEEN SYNCHRONY AND OUTCOME

Head-synchrony was strongly correlated with body-synchrony [*r*(69) = 0.40; *p* < 0.001], therefore associations between synchrony and outcomes were also calculated with the synchrony effect of the respective other ROI partialled out (see **Table 2**). The two sets of outcomes differ with respect to the time of assessment: micro-outcomes are obtained at the end of each session and relate to the current session only; macro-outcomes are assessed upon termination of therapy and relate to the whole course of treatment.

**Table 2:** Associations (Pearson’s *r*) between nonverbal synchrony and outcome.

Outcome variable	Head-synchrony	Body-synchrony	Head partial^1^	Body partial^1^	Head and body combined^2^
***Micro-outcome (at end of session)***
Alliance (Patient)	0.124	0.454***	-0.071	0.445***	0.407***
Self-efficacy (Patient)	0.292*	0.383**	0.164	0.304*	0.388***
Alliance (Therapist)	0.045	0.223^t^	-0.050	0.224^t^	0.257*
Patient’s resistance (Therapist)	0.007	-0.261*	0.126	-0.288*	-0.237*
***Macro-outcome (at termination of therapy)***
Goal attainment (GAS)	0.333**	0.145	0.300*	0.012	0.214^t^
Changes in experiencing and behavior (VEV)	0.261*	0.141	0.221^t^	0.036	0.158

^t^p< 0.10; *p< 0.05; **p< 0.01; ***p< 0.001.

*^1^Correlation between synchrony and outcome with other synchrony partialled out.*

*^2^Both regions combined (sum), i.e., whole-body movement.*

The synchrony-outcome associations indicated a differential pattern of relationships between head- versus body-synchrony and micro- versus macro-outcome: body-synchrony was associated with micro-outcome [*r*(69) = 0.22–0.45], whereas head-synchrony was to a lesser extent (*r* = 0.05–0.29). Head-synchrony was related to macro-outcome [*r*(69) = 0.26; 0.33], whereas body-synchrony was not [*r*(69) = 0.14; 0.15; see **Table 2** for details]. The most notable difference in associations between synchrony and micro-outcome was found in patient’s alliance and body-synchrony [*r*(69) = 0.45; *p* < 0.0001] and patient’s alliance and head-synchrony [*r*(69) = 0.12; *p* = n.s.]. A reversed pattern showed up in the association between synchrony and macro-outcome: goal attainment was associated with head-synchrony [*r*(69) = 0.33; *p* < 0.01], but not with body-synchrony [*r*(69) = 0.14; *p* = n.s.].

## DISCUSSION

Nonverbal synchrony is a pervasive phenomenon found in many different situations of human interaction. Building on previous findings in the psychotherapy setting (Ramseyer and Tschacher, 2008, 2011), the present extended analysis addressed the frequencies of head- and body-movement of patients and therapists. Females moved more than male participants in therapeutic dyads, and patients more than therapists. The main goal was to explore the relative contributions of head- versus body-synchrony to the embodiment of session-level assessments (micro-outcome) and global therapy success (macro-outcome). Using the sample of our previous study, we replicated the result that synchrony existed at a level above chance in both head- and body-synchrony. The associations with outcome were at levels equivalent to those previously found. Yet the present analyses uncovered additional patterns of associations with outcome indicating differential aspects of embodied phenomena: synchronized head-movement was associated particularly with the macro-outcome of psychotherapies, whereas synchronized body-movement predicted short-term micro-outcome at the session-level.

The differential contribution of head- and body-synchrony suggests that distinct aspects of embodied cognition may be effective in psychotherapy: the associations of body-synchrony with alliance found at the session-level may be interpreted as evidence for nonverbal signals that operate completely outside of conscious awareness, and thus may be more strongly associated with immediate effects on relationship quality and emotions. Movements of the torso and the changing of seating positions are processes that require little or no conscious deliberation (Dittmann, 1987), which makes them more susceptible to being automatically triggered in resonating individuals. The implicit association of body-movement with emotional processes is a possible example for this purported link: in therapy phases with high emotional activation, an example for such an emotion-regulation strategy is the changing of posture (Scheflen, 1964; Mehrabian, 1969), and at the level of gestures, the use of so-called self-adaptors (Barroso et al., 1978; Ulrich and Harms, 1985; Lausberg and Kryger, 2011) – gestures that are present in times of heightened emotional stress. The same would be true for gestures that accompany speech, especially in the case of so-called beat gestures – gestures with little or no semantic content (Wagner et al., 2014). Gestures have been shown to be synchronized in dialog (Kimbara, 2008; Holler and Wilkin, 2011). From the perspective of embodied cognition (Tschacher and Bergomi, 2011), the associations with patient-rated alliance and therapist-rated resistance would thus reflect the observable nonverbal manifestation of this immediate expression of therapeutic alliance, and possibly resonance in emotion-regulation, between patient and therapist. Therefore, the patient’s general impression of how helpful and how sympathetic the therapist has been in a session would thus be more closely reflected by the synchronized movements of the bodies, not the heads, of interacting persons.

The link with emotional, implicit content was less pronounced in head-movement synchrony: head movement is correlated with speech activity (Heylen et al., 2011) – e.g., nodding one’s head in connection with affirmative verbalisations – which is a more consciously controlled activity. Hadar et al. (1983) found a high proportion of head-movement (89.9%) during speech activity. A patient likely exerts more deliberate control over her/his head movement compared to her/his body-movement. Movements located more toward the periphery of the body are generally assumed to elude conscious control (Pease and Pease, 2006; Reiman, 2007; Goman, 2008). Head-movement synchrony should thus be more closely associated with long-term aspects of the patient–therapist relationship. This would be the case in a session where the patient experienced a lower alliance with the therapist, but where the overall therapy quality was favorable in a way that the patient “stayed in sync” with the therapist in terms of head-synchrony. Thus the level of head-synchrony – as a potential indicator of (verbal and explicit) agreement on treatment goals and overall relationship quality – should be associated with the overall success of therapy, which was true in our sample.

Patients who manage to resonate with the movements of the therapist (or therapists that manage to get patients to adapt a more healthy movement pattern), could thus profit more from the stronger (more stable) bond emerging between them. This would then be reflected by a more successful reaching of therapy goals.

### LIMITATIONS AND STRENGTHS

No set of specific *a priori* hypotheses had been generated, which implies that the present exploratory findings should be interpreted with caution. Nevertheless, they fit well with current knowledge on embodied processes in psychotherapy dyads and thus may serve as possible starting points for future research.

The data used in this study have the important advantage of having been monitored several years before the formulation or implementation of the nonverbal synchrony approach described here. Neither therapists nor patients had any awareness of the concept of nonverbal synchrony and its potential assessment by MEA. All shown motor behavior was thus completely uninfluenced by the research questions presented here.

### SUMMARY AND CONCLUSION

The present findings are in favor of fine-grained analyses of human movement. This analytic approach has been available for several years, yet its application was restricted to rather invasive procedures such as magnetic motion tracking or time-consuming rating techniques. Frame-differencing methods are increasingly available now, and we think that their ease of applicability and the potential for re-analyses of existing material clearly speak for a more wide-spread use. We hope that our present exploration encourages more research that would allow elaborating more and more differential methods that depict qualitatively distinct processes occurring in the domain of nonverbal movement. To cite Freud, we think that apart from the basic questions that may be answered with these tools, the results also offer promise for future use in clinical practice. “A path leads from identification by way of imitation to empathy, that is, to the comprehension of the mechanism by means of which we are enabled to take up an attitude at all toward another mental life” (Freud, 1955, p. 53).

## Conflict of Interest Statement

The authors declare that the research was conducted in the absence of any commercial or financial relationships that could be construed as a potential conflict of interest.
